# Simultaneous Celiac and Pancreaticoduodenal Artery Aneurysms in Median Arcuate Ligament Syndrome: Successful Long-Term Management via Staged Hybrid Treatment

**DOI:** 10.70352/scrj.cr.26-0021

**Published:** 2026-04-15

**Authors:** Shoji Takagi, Yoshifumi Mitani, Masaaki Akai, Motohiko Yamada, Tomihiro Fukushima, Nobuhisa Tajiri, Koji Nakanishi

**Affiliations:** 1 Department of Gastroenterological Surgery, Japanese Red Cross Okayama Hospital, Okayama, Okayama, Japan; 2 Department of Pain Medicine, Japanese Red Cross Okayama Hospital, Okayama, Okayama, Japan; 3 Department of Radiology, Kagawa Prefectural Central Hospital, Takamatsu, Kagawa, Japan; 4 Department of Cardiovascular Surgery, Japanese Red Cross Okayama Hospital, Okayama, Okayama, Japan

**Keywords:** celiac artery aneurysm, case report, embolization, median arcuate ligament syndrome, pancreaticoduodenal artery aneurysm, surgical decompression

## Abstract

**INTRODUCTION:**

Median arcuate ligament syndrome (MALS) is characterized by chronic mesenteric ischemia resulting from external compression of the celiac artery (CA). While rare, this compression can alter local hemodynamics, potentially leading to the formation of visceral artery aneurysms. Although pancreaticoduodenal artery (PDA) aneurysms are a known collateral complication, the concurrent presentation of a poststenotic CA aneurysm and a collateral PDA aneurysm is exceedingly rare. We report the long-term outcomes of a patient with MALS complicated by simultaneous aneurysms of both the CA and the PDA.

**CASE PRESENTATION:**

A 59-year-old woman was admitted with sudden, severe abdominal and back pain. Imaging revealed severe hook-shaped stenosis of the CA, a 15-mm poststenotic CA aneurysm, and a 7-mm inferior PDA aneurysm. Given the high risk of rupture, urgent endovascular coil embolization of the CA aneurysm was performed first. Although the pain initially improved, severe symptoms recurred, suggesting a neurogenic etiology involving celiac plexus compression. Nine days after embolization, the patient underwent laparoscopic median arcuate ligament release with celiac neurolysis, which provided immediate relief. However, residual stenosis of the CA subsequently caused recurrent ischemic pain. Percutaneous transluminal angioplasty (PTA) was then performed, fully restoring antegrade flow and resolving all symptoms. At the 8-year follow-up, the patient remained asymptomatic with sustained patency of the CA. Notably, the untreated PDA aneurysm regressed spontaneously from 7 to 2 mm on follow-up CT 2 months after hemodynamic normalization and remained stable thereafter.

**CONCLUSIONS:**

This report illustrates the efficacy of a staged hybrid strategy—consisting of aneurysm embolization, surgical decompression with neurolysis, and PTA—for managing MALS complicated by multiple aneurysms. The restoration of antegrade celiac flow was associated with the normalization of collateral hemodynamics, subsequent resolution of ischemic symptoms, and the long-term spontaneous regression of the collateral PDA aneurysm.

## Abbreviations

CA
: celiac artery
MALS
: median arcuate ligament syndrome
NRS
: numeric rating scale
PCA
: patient-controlled analgesia
PDA
: pancreaticoduodenal artery
PTA
: percutaneous transluminal angioplasty

## INTRODUCTION

MALS is a clinical entity caused by the external compression of the CA and the celiac ganglion by the median arcuate ligament.^1)^ While the classic presentation involves weight loss and postprandial abdominal pain, chronic compression can occasionally induce significant hemodynamic alterations, leading to rare vascular complications such as poststenotic CA aneurysms^2)^ or collateral PDA aneurysms.^3)^ Although PDA aneurysms are a recognized complication of MALS, the simultaneous occurrence of both a poststenotic CA aneurysm and a collateral PDA aneurysm is an exceedingly rare pathology. Successfully managing this complex condition requires a strategic approach that addresses the risk of aneurysm rupture, alleviates neurogenic symptoms, and corrects the underlying hemodynamic disturbance. Herein, we present a rare case of MALS complicated by concurrent aneurysms of the CA and PDA. The patient was successfully treated using a staged hybrid protocol comprising endovascular embolization, laparoscopic surgical decompression, and PTA. We highlight the durable long-term outcomes over an 8-year follow-up period, specifically noting the spontaneous regression of the untreated collateral PDA aneurysm following hemodynamic correction.

## CASE PRESENTATION

A 59-year-old female patient with no significant medical history presented to our emergency department with sudden, severe left back and abdominal pain. She rated the pain as 10/10 on the NRS, describing it as sharp and throbbing. On arrival, she was hemodynamically stable and afebrile but appeared in significant distress. Physical examination showed a soft abdomen without peritoneal signs. Laboratory investigations, including liver and pancreatic enzymes and inflammatory markers, were within normal limits. Analgesic treatment with loxoprofen (180 mg/day) and tramadol (100 mg/day) provided only partial relief, reducing the pain intensity to an NRS score of 4–5/10.

### Imaging findings

**Fig. 1:**
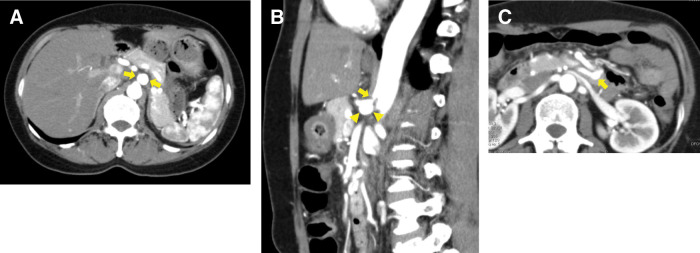
Preoperative contrast-enhanced CT findings. (**A**) Axial view showing a 15-mm CA aneurysm (arrows). (**B**) Sagittal view showing severe stenosis at the origin of the CA (arrow) and poststenotic aneurysm (arrowheads). (**C**) Axial view showing a 7-mm aneurysm of the inferior PDA (arrow). CA, celiac artery; PDA, pancreaticoduodenal artery

**Fig. 2:**
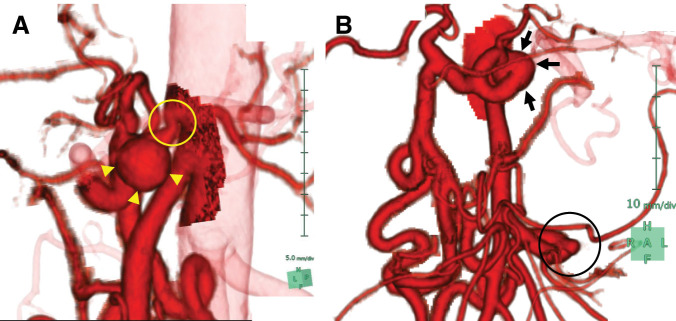
3D CT reconstruction findings at initial presentation. (**A**) Lateral view, confirming the hook-shaped stenosis (circle) and aneurysm of the CA (arrowheads). (**B**) Anterior view, showing the aneurysms of the CA (arrows) and inferior PDA (circle), with prominently developed collateral arcades from the superior mesenteric to the gastroduodenal arteries. CA, celiac artery; PDA, pancreaticoduodenal artery

Contrast-enhanced CT revealed severe hook-shaped stenosis at the origin of the CA, along with a 15-mm saccular aneurysm immediately distal to the stenosis and a 7-mm aneurysm in the inferior PDA (**Fig. 1**). Prominent collateral flow from the superior mesenteric artery to the CA through the pancreaticoduodenal arcade was observed. These findings were further supported by 3D CT reconstructions (**Fig. 2A** and **2B**). Doppler ultrasonography demonstrated a 15 × 13 mm CA aneurysm with a peak systolic velocity of 259 cm/s and reversed flow in the common hepatic artery, findings consistent with MALS.

### Clinical course and staged treatment

To clarify the complex clinical course and staged interventions, the timeline of events was as follows:

- Day 1: Admission with severe pain.
- Day 8: Urgent endovascular coil embolization of the CA aneurysm.
- Day 17: Laparoscopic median arcuate ligament release with celiac neurolysis.
- Day 31: PTA for residual stenosis.
- Day 32: Final discharge.

### Initial treatment: endovascular coil embolization

**Fig. 3:**
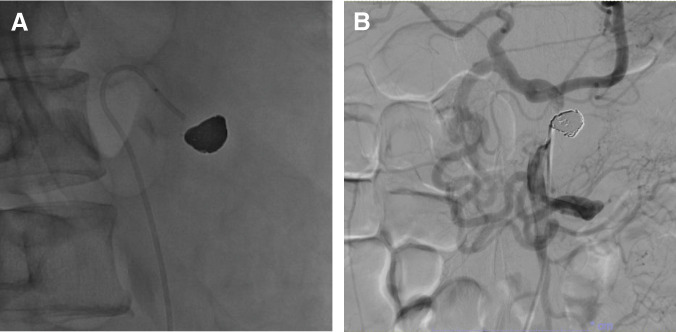
Interventional radiology findings during the initial embolization. (**A**) Coil embolization of the aneurysm of the CA using 7 Target XL coils (Stryker, Kalamazoo, MI, USA). (**B**) Angiography of the superior mesenteric artery performed after embolization. The gastroduodenal and common hepatic arteries were retrogradely visualized through the collateral pathways. CA, celiac artery

Given the high risk of rupture of the saccular CA aneurysm, urgent endovascular coil embolization was undertaken on hospital day 8. Vascular access was obtained via the right femoral artery. A 4-French shepherd hook guiding catheter was placed at the CA origin, and a 0.014-inch guidewire was carefully advanced across the severe stenotic segment. A microcatheter was subsequently introduced into the aneurysmal sac, and complete embolization was achieved using 7 Target XL coils (Stryker, Kalamazoo, MI, USA) (**Fig. 3A**).

Post-procedural angiography of the superior mesenteric artery demonstrated retrograde perfusion of the common hepatic and gastroduodenal arteries, consistent with persistent critical stenosis at the celiac origin (**Fig. 3B**). After embolization, the patient’s pain temporarily improved, with NRS scores ranging from 2 to 4. However, on hospital day 16, severe pain (NRS 10/10) recurred following defecation. Escalation of tramadol to 200 mg/day failed to achieve adequate pain control, necessitating intravenous morphine administration via PCA. One day after the recurrence of severe pain (9 days after embolization), a surgical intervention was performed.

### Surgical decompression and neurolysis

**Fig. 4:**
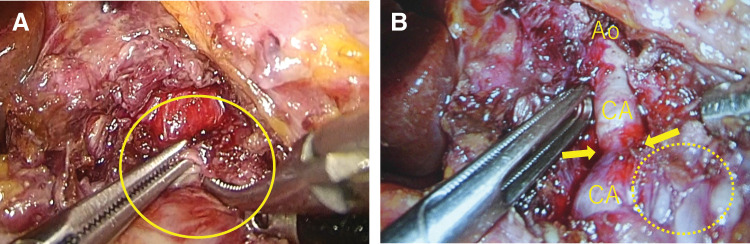
Intraoperative findings during laparoscopic release. (**A**) Identification of the median arcuate ligament (circle). (**B**) View following ligament release, with the Ao and CA exposed. Note the “waist” of the CA at the site of stenosis (arrows) and the poststenotic aneurysm (dotted circle). Ao, aorta; CA, celiac artery

On hospital day 17, laparoscopic release of the median arcuate ligament was undertaken. A transperitoneal approach was selected to provide adequate exposure of the ligament fibers and the celiac plexus. The procedure was performed under general anesthesia using a five-port technique with the patient in the lithotomy position. The liver was elevated with a Nathanson retractor. The diaphragmatic crura were dissected bilaterally to expose the anterior aspect of the aorta. The median arcuate ligament was identified as thickened and fibrotic, compressing the CA (**Fig. 4A**). Extensive dissection of the celiac plexus was carried out to expose the CA; however, dissection adjacent to the aneurysm neck on the left side of the CA was deliberately avoided to reduce the risk of rupture or bleeding (**Fig. 4B**). A cardiovascular surgical team was available on standby, and conversion to open surgery with vascular reconstruction was planned as a contingency in the event of intraoperative vascular injury. The operative time was 200 minutes with negligible blood loss. The patient reported immediate postoperative pain relief. Doppler ultrasonography performed on POD 1 demonstrated a bidirectional “to-and-fro” flow pattern in the common hepatic artery. Given the dramatic improvement in symptoms, the patient was discharged on POD 5.

### PTA for residual stenosis

**Fig. 5:**
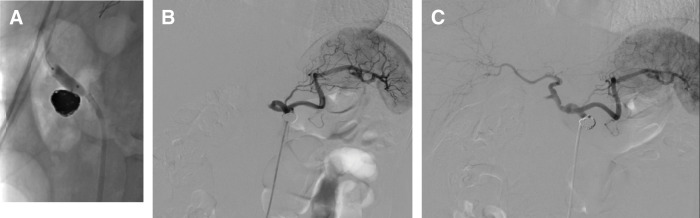
Percutaneous transluminal angioplasty and angiographic outcomes. (**A**) Fluoroscopy image showing that the stenosis of the CA was dilated using 5-mm balloon catheters, resulting in the disappearance of the stenotic notch. (**B**) Celiac angiography image captured before PTA, showing that the proper hepatic artery was not visualized. (**C**) Celiac angiography image captured after PTA, showing clear visualization of the proper hepatic and hepatic arteries and indicating a marked improvement in blood flow. CA, celiac artery; PTA, percutaneous transmunial angioplasty

On POD 8, the patient developed recurrent postprandial abdominal and back pain (NRS score, 4–5/10). Contrast-enhanced CT scan revealed residual CA stenosis. Consequently, she was readmitted on POD 14 after MAL release with neurolysis, and PTA was performed on the same day via right femoral artery access. Balloon dilation was initiated using a 3.5 mm × 2 cm balloon, followed by 2 inflations with a 5 mm × 2 cm balloon, each maintained at 8 atm for 3 minutes (**Fig. 5A**). Balloon sizing was determined according to the measured diameter of the common hepatic artery. Before angioplasty, the proper hepatic artery was not visualized on celiac angiography; following PTA, the proper hepatic artery and its distal branches became clearly opacified, with restoration of antegrade flow (**Fig. 5B** and **5C**). The patient’s postprandial pain resolved within 24 hours of the procedure. Doppler ultrasonography performed the following day demonstrated stable antegrade flow in the common hepatic artery, with a CA peak systolic velocity of 94 cm/s and no respiratory variation. The patient was discharged the day after PTA, and analgesic medications were gradually tapered and discontinued in the outpatient setting. The concomitant inferior PDA aneurysm was managed conservatively with close follow-up.

### Follow-up

**Fig. 6:**
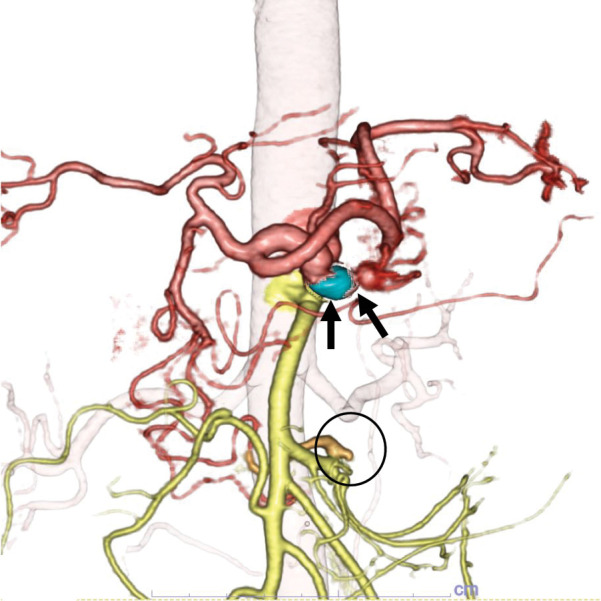
3D tomography scan captured at the 8-year postoperative follow-up showing regression of the collateral arcades. The CA aneurysm (arrows) remains embolized and stable, whereas the aneurysm of the inferior PDA (circle) has spontaneously regressed. CA, celiac artery; PDA, pancreaticoduodenal artery

The patient has been monitored regularly since treatment, with follow-up intervals gradually extended from every 3 months to every 6 months. At 8 years after intervention, she remains free of symptoms. Serial CT angiography demonstrated continued patency of the CA and stability of the coil-embolized aneurysm. Of particular note, the untreated inferior PDA aneurysm decreased in size from 7 to 2 mm 2 months after restoration of antegrade celiac flow, as documented on follow-up contrast-enhanced CT, and has remained unchanged in size over the subsequent 8 years. 3D CT imaging showed marked regression of the previously prominent collateral pathways (**Fig. 6**). Doppler ultrasonography findings have remained within normal limits, demonstrating stable antegrade flow in the common hepatic artery.

## DISCUSSION

This case represents an uncommon manifestation of MALS, characterized by abrupt severe abdominal and back pain and concurrent aneurysms of the CA and the inferior PDA. The condition was successfully treated using a staged hybrid approach that integrated endovascular intervention with laparoscopic surgery, resulting in sustained long-term clinical stability. A structured search of the PubMed database using the terms “median arcuate ligament syndrome,” “celiac artery aneurysm,” and “pancreaticoduodenal artery aneurysm” identified no previous reports describing this specific constellation of vascular lesions in a single patient.

The success of our staged protocol—embolization, surgical release, and PTA—validates the importance of sequentially addressing the distinct pathophysiological mechanisms of MALS. First, the initial embolization secured the CA aneurysm, eliminating the immediate rupture risk.^4,5)^ However, the recurrence of intractable pain despite successful embolization strongly indicated a neurogenic etiology. Subsequently, the immediate resolution of pain following laparoscopic decompression and neurolysis confirmed that celiac plexus compression was the primary source of symptoms. We selected a transperitoneal laparoscopic approach rather than a retroperitoneal approach because it offers superior visualization of the anterior aorta and celiac root, facilitating precise ligament release and neurolysis.^1,6–8)^ Furthermore, given the presence of the CA aneurysm, this anterior approach was deemed safer for directly identifying and preserving the aneurysm sac. Finally, the recurrence of postprandial pain indicated residual ischemia due to persistent stenosis and vessel remodeling.^9)^ The successful resolution of symptoms following PTA supports previous findings that while angioplasty alone is often ineffective for MALS, it serves as a crucial adjunctive therapy for residual stenosis following surgical release.^9–11)^ Retrospectively, earlier recognition of the hemodynamic symptoms might have theoretically allowed for earlier intervention. However, the residual ischemia was initially masked by the dramatic relief of neurogenic pain and only became evident after the patient resumed daily activities, thus justifying this stepwise approach.

The optimal management strategy for MALS-associated collateral aneurysms remains controversial. A fundamental question that has been the subject of considerable debate is whether to intervene directly on the aneurysm due to the risk of rupture, or to focus solely on hemodynamic correction. A critical decision in this case was the management of the collateral PDA aneurysm. Although vascular surgery guidelines^4)^ often recommend treating PDA aneurysms regardless of size due to their high rupture risk, we hypothesized that in the specific context of MALS, this aneurysm was a secondary hemodynamic consequence of collateral flow overload. Therefore, we prioritized correction of the upstream pathology (CA stenosis) to reduce collateral flow demand. This approach proved effective, as spontaneous regression of the aneurysm from 7 to 2 mm was observed on follow-up contrast-enhanced CT performed 2 months after restoration of antegrade celiac flow, a finding consistent with recent reports suggesting that correction of celiac hemodynamics alone can induce aneurysm regression.^12,13)^ This observation suggests that treating the primary hemodynamic abnormality in selected MALS cases may be sufficient to reverse secondary pathologies, thereby avoiding unnecessary and high-risk interventions on collateral aneurysms.

## CONCLUSIONS

We successfully treated a rare case of MALS with concurrent CA and PDA aneurysms using a staged hybrid strategy, achieving 8-year asymptomatic survival. Notably, spontaneous shrinkage of the collateral aneurysm following hemodynamic correction suggests that addressing the primary pathology may be an effective strategy for managing secondary aneurysms.

## DECLARATIONS

### Availability of data and materials

Data sharing is not applicable to this article, as no datasets were generated or analyzed during the current study.
